# Signalogs: Orthology-Based Identification of Novel Signaling Pathway Components in Three Metazoans

**DOI:** 10.1371/journal.pone.0019240

**Published:** 2011-05-03

**Authors:** Tamás Korcsmáros, Máté S. Szalay, Petra Rovó, Robin Palotai, Dávid Fazekas, Katalin Lenti, Illés J. Farkas, Péter Csermely, Tibor Vellai

**Affiliations:** 1 Department of Genetics, Eötvös Loránd University, Budapest, Hungary; 2 Department of Medical Chemistry, Faculty of Medicine, Semmelweis University, Budapest, Hungary; 3 Department of Morphology and Physiology, Faculty of Health Sciences, Semmelweis University, Budapest, Hungary; 4 Statistical and Biological Physics Research Group of the Hungarian Academy of Sciences and Eötvös University, Budapest, Hungary; Ecole Normale Supérieure de Lyon, France

## Abstract

**Background:**

Uncovering novel components of signal transduction pathways and their interactions within species is a central task in current biological research. Orthology alignment and functional genomics approaches allow the effective identification of signaling proteins by cross-species data integration. Recently, functional annotation of orthologs was transferred across organisms to predict novel roles for proteins. Despite the wide use of these methods, annotation of complete signaling pathways has not yet been transferred systematically between species.

**Principal Findings:**

Here we introduce the concept of ‘signalog’ to describe potential novel signaling function of a protein on the basis of the known signaling role(s) of its ortholog(s). To identify signalogs on genomic scale, we systematically transferred signaling pathway annotations among three animal species, the nematode *Caenorhabditis elegans*, the fruit fly *Drosophila melanogaster*, and humans. Using orthology data from InParanoid and signaling pathway information from the SignaLink database, we predict 88 worm, 92 fly, and 73 human novel signaling components. Furthermore, we developed an on-line tool and an interactive orthology network viewer to allow users to predict and visualize components of orthologous pathways. We verified the novelty of the predicted signalogs by literature search and comparison to known pathway annotations. In *C. elegans*, 6 out of the predicted novel Notch pathway members were validated experimentally. Our approach predicts signaling roles for 19 human orthodisease proteins and 5 known drug targets, and suggests 14 novel drug target candidates.

**Conclusions:**

Orthology-based pathway membership prediction between species enables the identification of novel signaling pathway components that we referred to as signalogs. Signalogs can be used to build a comprehensive signaling network in a given species. Such networks may increase the biomedical utilization of *C. elegans* and *D. melanogaster*. In humans, signalogs may identify novel drug targets and new signaling mechanisms for approved drugs.

## Introduction

Signal transduction pathways are involved in the control of various cellular processes, including cell growth, proliferation, differentiation and stress response in divergent animal phyla [1]. In humans, dysregulation of signaling systems has been implicated in diverse pathologies, such as cancer, neuronal degeneration, muscle atrophy, immune deficiency and diabetes [2]. To understand better the physiological and pathological roles of signaling pathways, one should generate a comprehensive signaling map (network) that ideally contains all components of distinct signaling pathways and their genetic and physical interactions. Currently, studies in model organisms ranging from invertebrates to mammals are increasingly used to create such a network [3]. The effort to map novel signaling components and interactions has largely benefited from network alignment techniques and other widely used functional genomics methods, allowing the integration of functional data both among and within species [4], [5]. For example, recent publications applied large-scale data integration and machine learning techniques to predict gene function, including signaling pathways in *D. melanogaster* [6], [7].

Most of these methods predict new gene or protein properties (annotations) on the basis of sequence homology and similarities between known functions. Similar annotation transfer approaches have been applied to predict structural properties (e.g., domain composition), expression profiles, and physical interactions for thousands of proteins [8]–[10]. For predicting interactions, for example, the network-based concept of “interologs” has been suggested: two proteins are predicted to physically interact, if their orthologs interact in another organism [11]. Interologs, however, were found to be less conserved than orthologs [12], and less reliable than interactions generated by high-throughput (HTP) approaches [13]. A clear definition of interologs and their applicability to estimate the reliability of HTP experiments [14], [15] have been found to be useful in expanding protein interaction networks [12], [16]–[18]. The concept of interologs has been extended to “regulogs”, which can be identified by an orthology-based prediction of a regulatory interaction between a protein (i.e., a transcription factor) and a corresponding DNA sequence (i.e., a transcription factor binding site). In addition, recently, “phenologs” were used as predictors of disease-associated genes in model organisms [19].

In large-scale analyses, protein-protein interaction data are usually obtained from HTP experiments, such as yeast two-hybrid screens. However, the low abundance of extracellular, membrane-bound and nuclear signaling components (e.g., ligands, receptors, and transcription factors) makes these experimental techniques only moderately efficient for identifying signaling interactions. Accordingly, several signaling pathway databases have been created manually by collecting relevant data from the literature [20]. However, so far most of them lack those key features (e.g., uniform pathway curation across more than one species) that would be necessary for transferring signaling pathway membership information between species. Reliable and detailed signaling pathway databases are crucial for signaling predictions because they are needed (1) as sources of known pathway information from which the predictions can be performed (i.e., seed data) and (2) as reference data sets against which the novelty of the predictions can be tested (i.e., those predicted signaling pathway member proteins that are already known pathway members should be removed from the list of predictions, while all others can be regarded as predicted components). Steps towards these goals have been taken, e.g., by Reactome [21], where pathway curation is standardized and human signaling functions are transferred from other species. Further steps could be (i) to compare predictions with already published experimental evidence in target species, (ii) to predict signaling reactions in other species, and (iii) to use a database that explicitly allows orthology-based predictions for pathway components between organisms. A recent, comprehensive pathway resource from our lab, SignaLink, applies uniform curation rules to keep the levels of details to be identical in all examined pathways for *C. elegans*, *D. melanogaster* and humans [22]. Moreover, the structure of the SignaLink dataset allows the systematic transfer of pathway annotations between two species on the basis of sequence orthology.

Interestingly, in two different organisms the same signaling pathway is often known at different levels of detail. This may be due to evolutionary divergence or to differences between the current coverage of the two organisms' interaction maps. Therefore, large-scale pathway annotation transfer between these 3 organisms can extend our current knowledge of their signaling pathways. Note that in cases of rapid evolution, orthology-based predictions are less reliable as even the orthologs exist, they no longer participate in the same signaling pathway [23].

The topology of signaling pathways is important for selecting possible novel drug target candidates [24]. As an example, drugs used for inhibiting a specific signaling protein in order to affect proliferation may actually activate the pathway by triggering an unknown negative feedback loop [25]. Transferring signaling pathway annotations across species may alleviate such difficulties and can provide a more comprehensive signaling network. Identification of novel signaling components may help to discover drug targets as (i) these signaling components can increase the applicability of model organisms for testing drugs and drug target candidates, (ii) in humans, they can serve as potential novel drug targets, and (iii) in the case of already used target proteins they can help to uncover possible side-effects.

Here we introduce the concept of ‘signalog’ to predict a protein as a novel component of a signaling pathway based on the signaling pathway membership of its ortholog in another organism. We identify signalogs on genomic scale in 8 signaling pathways, including the MAPK, TGF-β, and WNT pathways (for a complete list, see the Methods) from 3 intensively investigated species: *C. elegans*, *D. melanogaster* and humans, and verify their novelty and predictive power, using both bioinformatics and experimental methods. We also show the utility of the signalog concept in drug target discovery.

## Methods

### Source of signaling components and interactions

To predict a role for a protein (i.e. provide a successful annotation), the quality of the original sources from which the annotation transfer (prediction) can be performed is crucial. The original pathway data, including the lists of proteins of 8 distinct signaling pathways and their interactions were obtained from the SignaLink database (http://www.signalink.org) [22]. The 8 pathways examined in this study were the EGF/MAPK (epidermal growth factor/mitogen activated protein kinase), TGF-β (transforming growth factor-beta), insulin/IGF-1 (insulin-like growth factor-1), Notch, WNT/Wingless, Hedgehog, JAK/STAT (Janus activating kinase/signal transducer and activator of transcription), and NHR (nuclear hormone receptor) pathways. The basic properties of the SignaLink database are as follows: the components of the 8 pathways in *C. elegans*, *D. melanogaster*, and *H. sapiens* were compiled by applying uniform curation rules to keep the level of detail identical for all pathways examined; proteins were assigned to pathways based on literature data; and interactions were listed manually from original publications presenting biochemical evidence [22]. Note that the lower number of pathways in SignaLink, compared to other sources, is largely due to its more precise pathway definition rules. This approach avoids artificial grouping and reduces the number of pathways without reducing the numbers of proteins and interactions. (For an extensive comparison and benchmarking, see the [22].) Note that SignaLink uses more than 20 reviews per each pathway to list pathway components, in contrast to the average 5–15 reviews per a pathway in other resources [22]. The structure of SignaLink allows the systematic transfer of pathway annotations between two species on the basis of sequence orthology (see the next section).

### Orthology assignment for identifying signalogs

Sequence-based approaches, also in combination with interaction networks, have been frequently applied to detect orthology relationships between proteins [26], [27]. For example, the tool PathBLAST aligns an ordered list of proteins or pathways on the basis of their ortholog relations [28]. In the Clusters of Orthologous Groups (COG) database, orthologous groups are defined through reciprocal best BLAST matches between proteins from at least three species [29], [30]. Furthermore, sequence clustering techniques incorporate a range of BLAST scores (not only the absolute best hits) and can achieve a higher sensitivity [31]. One of these techniques, InParanoid [31], distinguishes between outparalogs, i.e., homologous sequences that emerged by duplication before speciation, and inparalogs that emerged after speciation. Compared to outparalogs, inparalogs are more likely to share functions. InParanoid incorporates the entire list of BLAST E-values (not only the top values) to group the proteins of the two compared organisms into orthologous clusters [31]. Each cluster contains proteins with related sequences from the two species, and each protein has an Inparalog score (for the calculation of this score, see [31]).

Previously, during the compilation of the SignaLink database, InParanoid data (version 6.1) were applied to find known signaling proteins by orthology searches [22], [31]. Based on SignaLink, now we can link a protein with a previously unknown signaling role to a signaling system, if the protein has an ortholog as a clearly identified component of a signaling pathway in another organism. For a protein with more than one ortholog (according to its InParanoid orthologous cluster), we used only those orthologs that have an Inparalog score higher than 0.3. To confirm that signalogs have no previously identified signaling interactions, we checked them with the protein-protein interaction search engines iHOP and ChiliBot [32], [33].

### Orthology-based pathway annotation transfer

**Figure 1:**
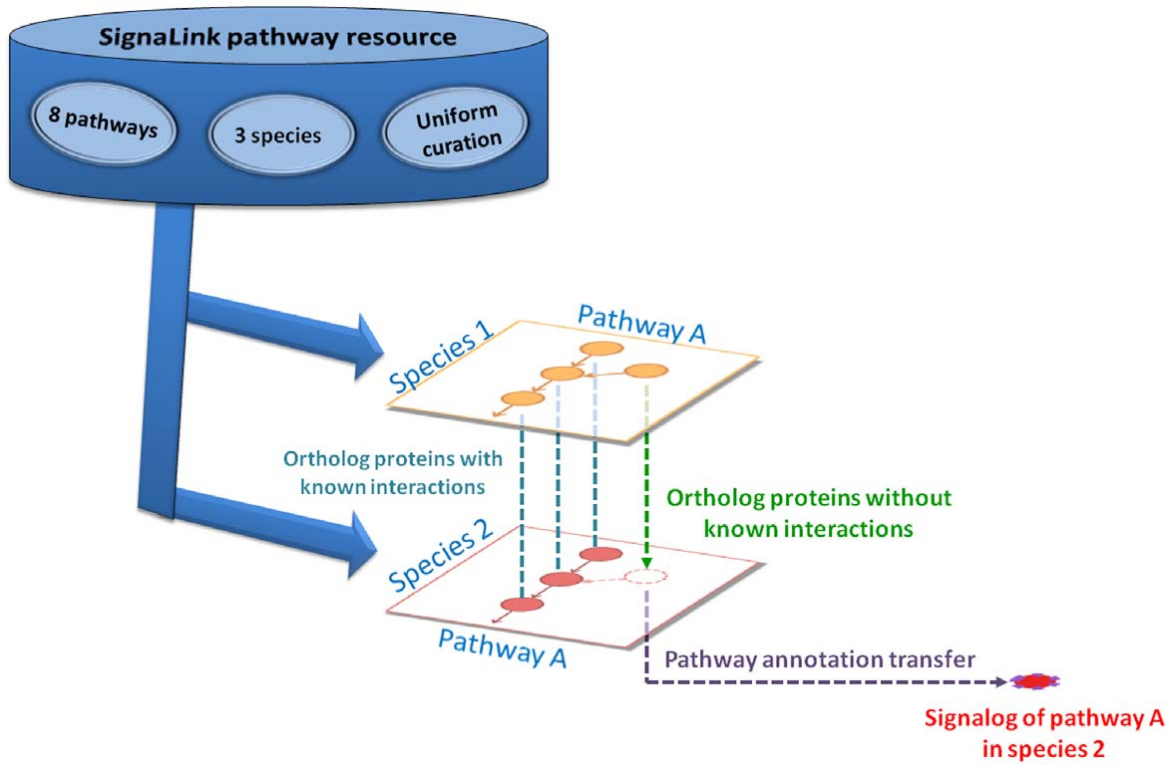
Workflow of pathway annotation transfer for predicting the signaling roles of proteins in *C. elegans*, *D. melanogaster*, and humans. The lists of signaling proteins and their interactions were obtained from the SignaLink database ([22], http://signalink.org). Orthology assignment was performed between each pair of the 3 species. Proteins were predicted to be members of the same signaling pathway(s) where their orthologs belong.

In each of the three species examined (*C. elegans*, *D. melanogaster*, and *H. sapiens*), we listed those proteins that have no known signaling interactions but have at least one signaling pathway member ortholog in the other two species. Similarly to the concept of functional orthology [26], for each of these proteins we assumed that their pathway annotations (i.e., signaling role) can be transferred between species. In other words, we predicted that such a protein is a member of the signaling pathway(s) to which its ortholog(s) in the organism belong(s). These proteins were termed as *signalog proteins* (*signalogs*). Note also that in SignaLink [22] a protein can belong to more than one pathway. Thus, a signalog can also be annotated to more than one pathway. Figure 1 shows the workflow of our analysis.

### Verifying the novelty of signalog predictions

To verify the novelty of signalog predictions, i.e., the predicted signaling roles have not been known or predicted in other resources yet, we have (i) searched the literature with semi-automated methods for already known annotations, (ii) compared the list of signalogs and their predicted pathway memberships to known pathway annotations in pathway databases, and also (iii) compared the ortholog predictions to previously published interolog predictions.

To grade the novelty of signalogs (signaling pathway annotations) and quantify the confidence level of each prediction, we performed semi-automated searches using PubMed, UniProt, GO, Wormbase, FlyBase, iHOP, and Chilibot web services [32]–[37]. During this process, direct manual curation and Python scripts checking multiple proteins in one webservice were used. In each of the 3 species examined, we classified the predicted signalogs into 5 groups on the basis of their known properties in the literature: (1) no orthology information and/or no biochemical function is available; (2) there are known orthologs with unknown biochemical function; (3) orthology information: unknown, biochemical function: known; (4) known orthologs with known biochemical function(s); (5) known orthologs with known biochemical function and already known pathway annotation(s). Categories 1 to 5 denote a decreasing level of novelty. However, even category (5) contains signalogs for which at least one novel signaling pathway membership is predicted. Furthermore, to check the novelty of the predicted signaling pathway memberships, we compared the list of signalogs and their predicted pathway memberships to known pathway membership annotations from Reactome and KEGG [21], [38].

Next, we applied interologs to verify the novelty of our ortholog predictions. (An interolog is a pair of proteins predicted to interact based on the interaction of the two proteins' orthologs in at least one other organism [11].) To reveal the presence of signalogs in current orthology-based prediction databases, we compared already identified interologs in worms, flies, and humans using 3 species-specific datasets (WI8, DroID, and HomoMINT) [12], [39], [40] with interologs generated from SignaLink data. Neither SignaLink [22] nor the current signalog identification approach identify interologs directly, thus we used an indirect method. First, we deduced interologs from SignaLink data by linking two proteins in an organism, if their orthologs interact in at least one other of the three organisms. After generating all possible interologs from SignaLink, we examined only those interologs (predicted interactions) in which at least one of the interactors is a signalog protein (predicted signaling pathway member).

### Experimental validation of signalogs

We confirmed experimentally the predicted signaling pathway memberships (signaling roles) of 6 signalogs. Out of the 21 Notch signalogs in *C. elegans*, we selected 6 genes (*aqp-6*, *D1009.3*, *nsh-1*, *num-1*, *F10D7.5* and *crb-1*) that have no paralogs, i.e., homologs in the same organism. These genes encode diverse proteins: their orthologs in the other two species include receptors, co-factors, and transcription factors (Table 1). Neither literature search (in PubMed) nor interaction searches in STRING 8.0 [14] provided experimental evidence for the signaling role of these proteins in the *C. elegans* Notch pathway. To validate that the selected 6 signalogs indeed function in the Notch signaling pathway, we tested whether they genetically interact with *lin-12*, which encodes a worm Notch receptor [41].

**Table 1:** Experimentally validated signalogs and their orthologs.

Selected signalogs (gene name)	ORFs (WormBase ID)	Mutant allele	Signalog class	Known signaling ortholog in fly	Known signaling ortholog in human	Role of the ortholog in the Notch pathway
*aqp-6*	*C32C4.2*	lf mutant: *(tm2407)V*	3	*bip*	-	co-factor
*crb-1*	*F11C7.4*	lf mutant: *(ok931)X*	3	*crb*	NOTCH2	receptor
*D1009.3*	*D1009.3*	lf mutant: *(ok1349)X*	1	*sca*	-	co-factor
*F10D7.5*	*F10D7.5*	no characterized mutant alleles	4	*neur*	NEURL	transcription factor, co-factor
*nsh-1*	*F20H11.2*	no characterized mutant alleles	2	*sno*	SBNO1	transcription factor
*num-1*	*T03D8.1*	lf mutant: *(ok433)V*	4	*numb*	NUMB	co-factor

We experimentally validated 6 novel Notch pathway components in *C. elegans*. 4 of them have been characterized by at least one loss-of-function mutant allele. None of these mutations caused altered morphology, behavior, or survival, i.e., these mutant animals appear superficially wild-type. Fly and human orthologs are also listed. Signalogs are classified into 5 groups (signalog classes) based on their known properties (see Methods and Fig. 3a). In the SignaLink database, each protein is annotated with one or more pathway role (e.g., co-factor, receptor).

Wild-type strain was used as Bristol N2 [42]. The other strains used in this study were RB676 *num-1(ok433)V*, FX02407 *aqp-6(tm2407)V*, RB1267 *D1009.3(ok1349)X*, RB1011 *crb-1(ok931)X*, and MT2343 *dpy-19(e1259)III lin-12(n137)III*/*unc-32(e189)III lin-12(n137n720)III*. The allele *n137* is a gain-of-function mutation in *lin-12*, which confers ectopic vulval induction in mutant hermaphrodites. These *lin-12(n137)* mutant animals exhibit a Multivulva (Muv) phenotype (the mutants possess 2–6 vulval protrusions, whereas wild-type hermaphrodites have normally one vulval structure, which does not protrudes from the ventral surface of the body). Strains were cultured as described previously [43]. For the selected genes that are not yet characterized mutationally and for *lin-12*, we applied RNA interference- (RNAi) based gene downregulation. For RNAi, 0.9 kbp (*nsh-1*) - 1.0 kbp long (*F10D7.5*) cDNA fragments were amplified by RT-PCR, and were cloned into the vector pPD129.36. The constructs were then transformed into *E. coli* HT115. RNAi experiments were performed as described [43]. Control strains were fed with HT115 bacteria expressing the empty RNAi vector. The following forward and reverse primers were used. For *nsh-1*: 5′-GGC TTT AAT GGC TTC ACG AG-3′ and 5′-AAG GAA GAA CTG TCG CTT GC-3′, for *F10D7.5*: 5′-AAG CGT TGA TCC GTA GAC ATC-3′ and 5′-TCG AGA TTG ACG AGA ACG TG-3′, and for *lin-12*: 5′-CGC TTC ATA TTG GCT CAT GTC-3′ and 5′-CCA GCT TCG CAT TTA TTA TTC AC-3′.

As *num-1(ok433)*, *aqp-6(tm2407) D1009.3(ok1349)*, and *crb-1(ok931)* single mutant animals looked superficially wild-type, we treated these mutant animals with *lin-12* double-stranded RNA (dsRNA), and the average number of vulval protrusions were determined at their young adulthood. *lin-12(gf)* mutant animals were also treated with *nsh-1* or *F10D7.5* dsRNA, and tested for the penetrance and expressivity of the Muv phenotype. The significance of results was tested with Chi square tests.

### Functional annotation of signalog proteins

To examine the drug target relevance of the predicted signaling pathway member proteins, we downloaded additional information from DAVID [44], and disease-related data from OMIM, GAD and Orthodisease [45]–[47]. Protein domain information was extracted from InterPRO (drug-protein interactions are based on structural properties) [48]. Functional and cellular compartment data were obtained from GO [49]. The list of currently used drug targets was downloaded from DrugBank (version 2.5) [50].

## Results

### Computational prediction and analysis of novel signaling components

We identified novel signaling pathway components based on the signaling pathway memberships of orthologs in another organism. We found 88, 92, and 73 proteins in *C. elegans*, *D. melanogaster* and *H. sapiens*, respectively, which had previously not been assigned to a signaling system, but have at least one ortholog in the other two species that is clearly associated with a signaling pathway. We hypothesized that these 253 proteins function in the same signaling pathways as their orthologs. Thus, we named the predicted signaling components signalog proteins, or briefly signalogs. Note that in contrast to an interolog, a signalog is a single protein (not an interacting pair) that has an ortholog annotated to a signaling pathway. In the three species examined our *in silico* approach predicted 301 novel signaling pathway annotations in total (39 of the 253 signalogs were assigned to more than one signaling pathway). For the complete list of the predicted signalog proteins and their pathway annotations, see Table S1.

**Figure 2:**
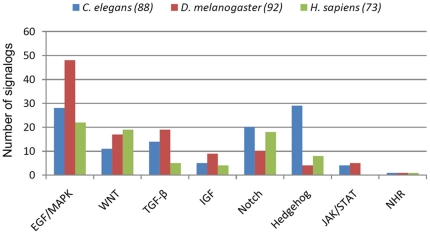
Statistical analysis of signalogs. The number of predicted novel signaling pathway components (signalogs) in each pathway for the three species examined. The total number of signalogs in each species is shown in parentheses. Although we predicted a total of 253 signalogs, this statistical comparison contains 301 novel pathway components, because 39 signalog proteins were assigned to more than one pathway (these 39 had a total of 87 pathway annotations).

After a detailed analysis of the predicted pathway annotations of signalogs, we found that in each of the three species the EGF/MAPK pathway contains the largest portion of signalogs (25% of the signalogs in the in worm, 42.5% in the fly and 28.6% in humans), which is consistent with the fact that this pathway contains the highest number of signaling components [22] (see also Fig. 2). Interestingly, in *C. elegans* a similarly high number of signalogs was predicted as a potential Hedgehog component (29 proteins; 25.9%). Note that a canonical Hh pathway has not been identified in *C. elegans* due to specific gene loss [1]. Our present study and previous analyses however show that several components of the canonical Hedgehog pathway are present in this organism [1]. In flies a large portion of signalogs appears in the WNT and TGF-β pathways, whereas in humans, several signalogs were associated with the WNT, Notch and Hedgehog pathways (for details, see Fig. 2). Beside pathway membership predictions, we placed the signaling orthologs into a wiring diagram of signaling networks. This network can be examined with an interactive ortholog network viewer (described below).

Next, we tested the concept of orthology-based pathway annotation transfer by testing whether known signaling proteins have known signaling pathway member orthologs. We analyzed the three organisms separately. For each organism we listed all signaling pathway proteins from SignaLink and then listed, with InParanoid (version 6.1) [31], all orthologs of these signaling proteins in the other two species. We found at least one ortholog in the other two species for 93.4% (worm), 81.6% (fly), and 64.8% (humans) of all signaling proteins and we found that 83.2%, 67.5%, and 82.6% of these orthologs indeed participate in a signaling pathway. Thus, a high portion (77.8% on average) of known signaling proteins has at least one ortholog with a known signaling function in the other two organisms. Moreover, on average 67.8% of signaling pathway member proteins have at least one ortholog in the other two species with an identical pathway membership. These high ratios underscore the relevance of our orthology-based signaling component prediction.

### Verifying the novelty of predictions

**Figure 3:**
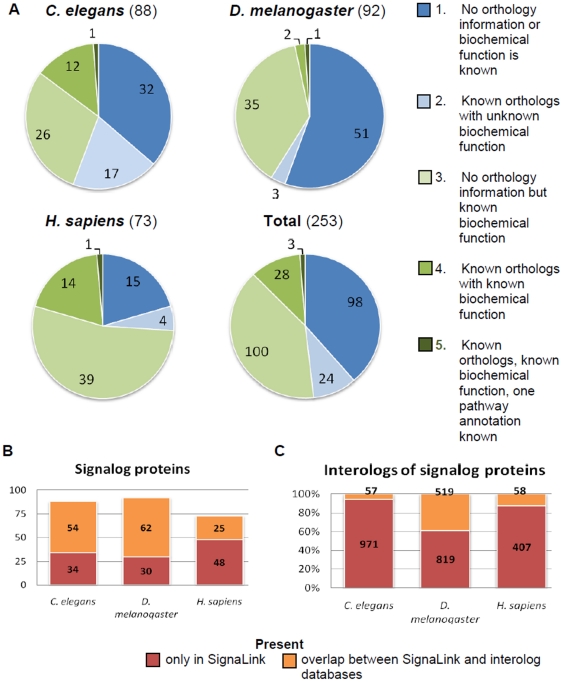
Verification of the novelty of signalogs (protein – signaling pathway annotations). a) Classification of signalog proteins based on their known properties in the literature found by manual curation and searches in PubMed, UniProt, GO, Wormbase, Flybase, iHOP, and Chilibot [32]–[35]. b–c) Comparison of signalog and interolog data between Signalink and one of the WI8, DroID or HomoMINT databases. The signalog proteins and possible interologs that are only present in SignaLink have not been predicted by previous orthology based (interolog) studies. This underscores the novelty of the signalogs. For details on interologs predicted from SignaLink data, see the main text.

To examine the novelty of the predicted signaling roles and to quantify their confidence levels, we first classified these predicted proteins into 5 groups on the basis of their known properties. These groups range from genes for which only the ORF is known to genes whose protein products have known molecular function(s) (for details see the Methods and Fig. 3a). In each of the three organisms examined, we found only one protein already known as a signaling pathway component; for these three proteins we predicted additional (novel) pathway annotations. In *C. elegans* and *D. melanogaster*, most signalogs (55.7% and 58.7% of all, respectively) have not yet been characterized biochemically, while in humans, only 26% of the signalogs remain uncharacterized. Note that this lower rate is partly due to the larger abundance of literature information on signaling in humans compared to worms and flies (Chi square test, p<0.0001). Taken together, we conclude that signalog prediction can effectively contribute to the identification of novel signaling components.

Next, we verified that our signolog predictions are novel in the sense that the predicted signaling roles have not been known or predicted by other resources yet. We compared the list of signalogs and their predicted pathway memberships to known pathway membership annotations from KEGG and Reactome databases. Note that the KEGG database contains pathway data for all three species examined, while Reactome database contains only human data [21], [38]. For *C. elegans*, *D. melanogaster* and *H. sapiens*, we found 23, 19 and 35 signalog proteins, respectively, already present in KEGG, i.e., 20%, 26%, and 47% of all signalogs in the three organisms. From these proteins only 11, 5 and 15, respectively – i.e., 13.3% on average – were assigned to the same pathway in KEGG as in our prediction. Reactome contains 16 of the listed human signalogs (22% of all), but only 1 in the same pathway, as in our prediction. We conclude that, depending on the organism and the database, 20 to 47% of all signalog proteins are already present in KEGG or Reactome, however, the large majority (86.7%) of pathway memberships we predicted for them are novel.

Finally, to reveal the presence of signalogs in current orthology-based prediction databases, we used interologs (orthology-based predicted interactions). We compared the interologs generated for this test from the SignaLink dataset with the interologs listed for worms, flies and humans by 3 species-specific databases (WI8, DroID, and HomoMINT [12], [39], [40]) (see the Methods). We examined only those interologs, where at least one of the interactors is a signalog protein. We found that in worms, flies and humans, respectively 34, 30, and 48 signalogs are present only in the SignaLink dataset,indicating that a high portion of the predicted proteins has not yet been investigated by this orthology-based prediction method (Fig. 3a). Altogether, in SignaLink and in the 3 species-specific resources, we found 1028, 1338, and 465 interologs in worms, flies and humans, respectively. The overlap between interologs generated from SignaLink and the interologs from any of the other 3 databases was relatively low: 5.5% in worms, 38.8% in flies and 12.5% in humans (Fig. 3b). Shared interologs can be interpreted as already known orthology-based predictions. A low number of overlapping interologs suggests that most of our current signalog predictions are novel. The high number of novel interologs is probably due to (i) the uniform curation method used for compiling SignaLink for all three species; (ii) the underrepresentation of signaling proteins in the three species-specific interolog datasets [5% of all signaling proteins in worms (WI8) and flies (DroID), and 2.8% in humans (HomoMINT)]; and (iii) the stringency of the interolog filtering algorithm HomoMINT [12]. We conclude that uniformly curated data sources, such as SignaLink, can facilitate orthology-based predictions.

While this study was in progress, Yan *et al.* predicted gene function for *Drosophila* with a large-scale machine learning technique [6]. 1121 genes were predicted to function in the same pathways as those examined in our study. We performed an additional test with this fly-specific dataset to quantify the novelty of our predictions. (Note that Yan *et. al.* [6] listed the ErbB, JNK, and MAPK pathways separately, following KEGG [38], however, in SignaLink these (sub)pathways all belong to the EGF/MAPK pathway in agreement with Ref. [1]). Out of the 92 fly signalogs predicted by the current paper, only 27 genes are listed in this large-scale study, i.e., two-thirds of all fly signalogs still remain novel predicted signaling components. In the large-scale study of Yan *et. al.* these shared 27 genes have 42 pathway annotations (many of them belong to more than one pathway), while here we predict 34 pathway annotations for them. 15 out of these pathway annotations are identical (for 14 of the 27 shared genes). Interestingly, a large-scale study predicted that *p38b* functions in 2 pathways (EGF/MAPK and TGF), while in our study 2 additional pathways (JAK/STAT and WNT) were predicted for this gene. In conclusion, the small overlap of the predicted novel signaling pathway genes and their predicted pathways verifies the novelty of the signalogs listed in the current paper and the predictive power of the method.

### Experimental validation of Notch pathway member signalogs in *C. elegans*

The Notch pathway controls cell growth, differentiation and proliferation during normal animal development [51]; in humans, aberrant Notch signaling has been implicated in various pathologies such as cancer and neurodegeneration [52]. Therefore, identifying novel Notch pathway components may have a significant impact on developmental and biomedical research. To test experimentally the relevance of our signalog predictions, we assessed whether the genes that encode the 6 newly identified non-paralogous Notch pathway components in *C. elegans* (*aqp-6*, *D1009.3*, *nsh-1*, *num-1*, *F10D7.5* and *crb-1*) genetically interact with *lin-12*, which encodes a nematode Notch receptor (see Table 1 for further information on the selected 6 genes). *lin-12* is a key regulator of vulval patterning as it specifies the so-called (2°) secondary vulval cell fate during pattering of this tissue [53]. Thus, a genetic interaction between *lin-12* and a selected signalog (gene) would clearly indicate the participation of that gene in Notch signaling.

**Figure 4:**
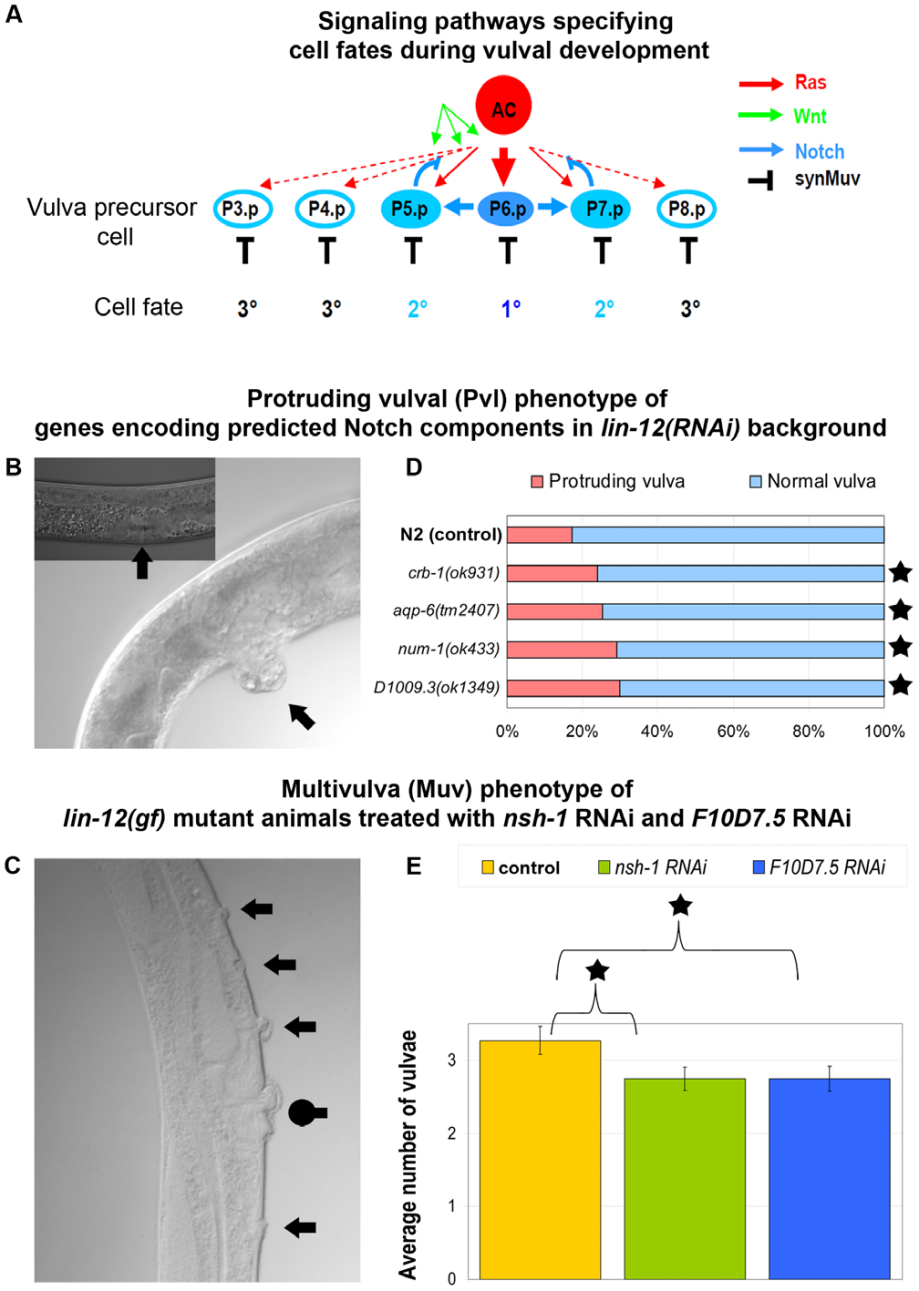
Experimental validation of 6 Notch pathway member signalogs in *C. elegans*. a) During normal *C. elegans* vulval development, the pattern of vulval precursor cells is determined by the combined action of distinct signaling pathways, including the Ras/MAPK, Wnt, Notch, and synMuv systems. Activations and inhibitions are represented with normal or blunted arrows. AC: anchor cell. Ras signaling activity is graded along its relative distance from the AC cell (displayed with thick, thin, and dotted red lines). b) Protruded vulva (Pvl, main panel) and wild-type vulva (N2) (inset). c) Ectopic vulval protrusions (arrows) and a normal vulval structure (dotted arrow) in a Muv animal. d) Penetrance of Pvl and normal vulval phenotypes in loss-of-function mutant animals treated with *lin-12* RNAi. In each case the mutation significantly increases the penetrance of Pvl phenotype. e) Average number of vulval inductions in *lin-12(gf)* mutant animals treated with *nsh-1* or *F10D7.5* RNAi. Observe that both *nsh-1 RNAi* and *F10D7.5 RNAi* have smaller numbers of vulvae than the control strain, *lin-12(gf)*. Asterisks denote statistically significant differences. For details of the statistics, see Text S1.

During *C. elegans* vulval development, six originally equivalent ventral epidermal cells, called vulval precursor cells [VPCs, consecutively numbered as P(3–8).p], are specified into one of three distinct – primary (1°), secondary (2°) or tertiary (3°) – vulval cell fates by the combined action of different signaling systems, including the Ras/MAPK, WNT, Notch and synMuv (for synthetic Multivulva) pathways [54] (Fig. 4a). Recently, the nematode sex determination pathway was also implicated in vulval fate determination [55]. At the L3 larval stage, the inductive Ras and WNT signaling cascades promote vulval fates in the three central VPCs, P(5–7).p; descendants of these cells eventually form the matured vulval tissue. A LIN-12/Notch-mediated lateral signal emitted from P6.p specifies 2° fates by attenuating Ras signaling in the adjacent VPCs, P(5,7).p [41], [56]. An inhibitory signal mediated by the *synMuv* genes antagonizes Ras signaling to repress vulval fates in each VPC [57]. As a result of these inductive, lateral and inhibitory signaling events, P6.p adopts a primary (1°) vulval fate, while its adjacent VPCs, P(5,7).p, adopt secondary (2°) vulval fates. The non-induced VPCs, P(3,4,8).p, adopt non-vulval tertiary (3°) fates. In wild-type hermaphrodites, P(3–8).p always adopt the 3°-3°-2°-1°-2°-3° stereotypical pattern of vulval fates.

Perturbation of signals specifying vulval fates often results in a visible mutant vulval phenotype. Inactivation of *lin-12* causes a Protruded vulva (Pvl) phenotype that is due to the misspecification of 2° vulval cells; in *lin-12* loss-of-function (lf) mutant hermaphrodites each induced VPC [P(5–7.p] adopts a 1° fate (Fig. 4b), whereas *lin-12* gain-of-function (gf) mutations confer VPCs [P(3–8).p] to adopt 2° fates, resulting in multiple vulval protrusions, i.e., a Multivulva phenotype (Muv) (Fig. 4c).

To reveal whether the 6 selected *C. elegans* Notch signalogs (Table 1) indeed function in this pathway, we first treated *aqp-6*, *crb-1*, *num-1*, and *D1009.3* loss-of-function mutant animals with *lin-12* dsRNA, and monitored the penetrance of the Pvl phenotype, compared to control *lin-12(RNAi)* animals. We found that *lin-12* RNAi treatment of wild-type animals cause a Pvl phenotype with 17% penetrance. As compared, *lin-12* RNAi in *aqp-6*, *crb-1*, *num-1*, and *D1009.3* single loss-of-function mutant background significantly increased the penetrance of the Pvl phenotype (Fig. 4d). Note that these single mutants treated with control RNAi (empty vector) displayed a superficially wild-type (non-Muv) vulval morphology.

We next treated *lin-12* gain-of-function mutant animals with *nsh-1* or *F10D7.5* dsRNA (these two genes have not yet been characterized by mutant alleles) and monitored the average number of vulval protrusions, compared to those found in *lin-12(gf)* animals expressing the control RNAi alone. Of these *lin-12(gf)* mutants, 93% were Muv and the number of vulval inductions was 3.27+/−0.19. The penetrance and expressivity of the Muv phenotype in *lin-12(gf)* mutants was reduced by *nsh-1* or *F10D7.5* RNAi treatment. Depletion of NSH-1 decreased the penetrance of the Muv phenotype to 83%, whereas silencing of *F10D7.5* reduced it to 81%. Both RNAi interventions significantly reduced the effect of *lin-12* hyperactivity on vulval induction (Fig. 4e). In summary, we found that all 6 selected genes significantly alter vulval induction in *lin-12(RNAi)* animals and *lin-12(gf)* mutants (for results, see Fig. 4; for statistics, see Text S1). Thus, all 6 genes genetically interact with *lin-12* and may participate in Notch signaling. Note that these genetic interactions between the 6 tested genes and the worm Notch receptor may be indirect. Further, in depth biochemical studies are needed to uncover the details of these connections, the actual roles of the 6 encoded proteins in the Notch pathway, and their roles in other pathways involved in vulval formation.

### On-line prediction and visualization tool for orthologous signaling networks

To facilitate the adaptability of the signalog prediction concept, we developed an on-line tool. This tool is available at http://signalink.org/signalog and performs the same workflow that was presented in this study. After selecting the target species of our prediction (worm, fly, or human), the user can enter a search term (arbitrary protein or gene name/ID). The on-line tool understands a variety of names and IDs, and also name fragments. Next, an integrated UniProt API synonym search helps the user to select the actual protein [34]. The selection of the protein is followed by an ortholog search in the InParanoid database [31], and the list of known orthologs is listed for the other 2 species. Next, the pathway memberships of the orthologs are shown, and the user can select a pathway of interest. Currently, the tool uses only SignaLink pathway data. If other sources become available with uniform pathway curation across all curated species and pathways, which is crucial for proper pathway annotation transfer between species, the on-line tool is capable to include these resources as well. In the last step, a pathway membership prediction is performed for the queried protein. If the queried protein is not known to be a member of the examined pathway, then it is a signalog of that pathway. The result is presented with a confidence score and a newly developed visualization tool.

**Figure 5:**
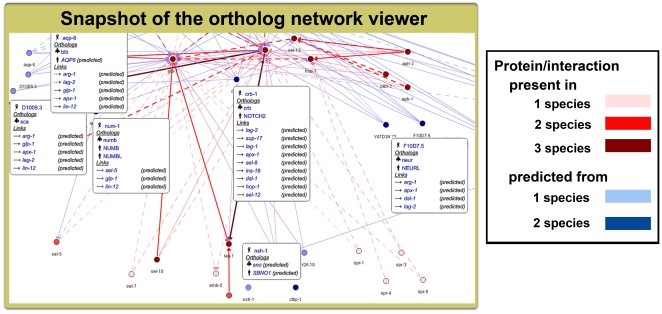
Snapshot of the Notch pathway visualized with the ortholog network viewer. This figure displays a subset of the *C. elegans* Notch pathway with selected predicted pathway member proteins and their properties. This image includes multiple snapshots taken from the interactive ortholog viewer available at http://signalink.org. Links representing interactions with direct and indirect evidence are displayed as normal and dashed line styles, respectively. Activations (and inhibitions) are represented with normal (and blunted) arrows.

Despite the relatively large number of currently available interaction visualization tools [58], only few visualize known and predicted proteins and/or interactions in parallel. The viewer of the MINT database is one such example [12]. This viewer displays interactions predicted from model organisms, but not from humans. Note that MINT [12] is a general protein-protein interaction database containing significantly less signaling pathway information than analyzed here. We developed an interactive ortholog network viewer available at http://signalink.org and http://signalink.org/signalog. This viewer can simultaneously visualize known and predicted pathway membership information, i.e., pathway annotation transfers (see snapshot in Fig. 5 as an example), and allows the user to analyze, individually or together, the examined 8 signaling pathways in the 3 species. Interactive features of this signaling network viewer include zooming in/out and panning possibilities, and switching the pathway view between organisms. Proteins are hyperlinked to species-specific databases and interactions are hyperlinked to the PubMed abstract of the article(s) providing experimental evidence. Known signaling pathway member proteins and signaling interactions are visualized with colors different from predicted ones.

### Supporting drug target discovery with signalogs

Signaling proteins are overrepresented among human disease genes [2] and have been intensively studied as potential drug target candidates [22], [59]. According to DrugBank [50], only 5 (6.8%) out of the 73 human signalog proteins identified here are currently considered as drug targets (Table 2). Interestingly, the ratio of disease-related proteins among human signalogs is much higher: 18 out of 73 (24.6%). The remaining 68 human signalog proteins not yet implicated as drug targets may serve as further candidates (Table S2).

**Table 2:** Novel signaling roles of signalogs relevant for drug target discovery.

Signalog	Predicted signaling pathway	Known as the target of the following drug(s)	Drug target-like relevance score
ANPRA	WNT	Erythrityl Tetranitrate, Isosorbide Dinitrate, Isosorbide Mononitrate, Nesiritide, Nitric Oxide, Nitroglycerin, Nitroprusside	4
CASK	EGF/MAPK	Formic Acid	4
INSRR	IGF/insulin	-	3
MARK2	EGF/MAPK, WNT	-	3
ABL2	Notch	Adenosine Triphosphate, Dasatinib	2
AP2A2	Notch	-	2
AQP8	Notch	-	2
AUXILIN	Notch	-	2
B3GL1	Notch	-	2
BMP2K	Notch	-	2
EDD1	Hedgehog	-	2
FZD6	WNT	-	2
GNAZ	EGF/MAPK	-	2
PIK3R2	IGF/insulin	-	2
PTPRB	EGF/MAPK	-	2
STK24	WNT	-	2
UGDH	Hedgehog	Nadide	2
YWHAB	WNT	-	2
NDST1	Hedgehog	Adenosine-3′-5′-Diphosphate	1

**Figure 6:**
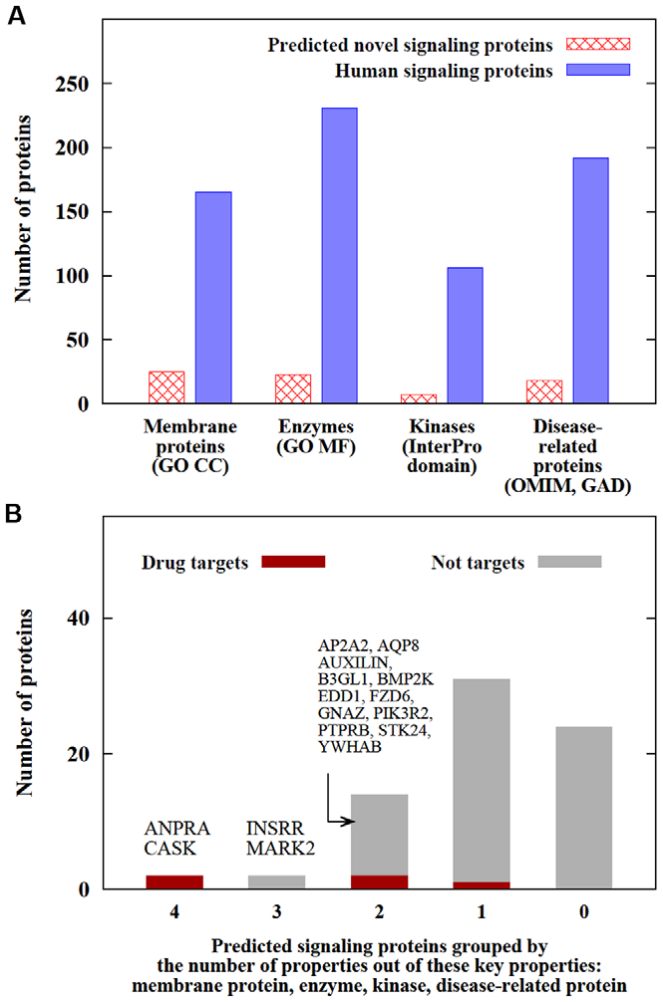
Analysis of signalogs as drug target candidates. a) Numbers of membrane proteins (M), enzymes (E), proteins with a kinase domain (K), and disease-related proteins (D) among signalog proteins (control: all proteins of the SignaLink database). b) Numbers of signalogs in the groups defined by the number of key properties out of the listed 4 (M, E, K, and D). For each group, the number of drug targets is shown separately. The names of the most promising drug target candidate proteins and 2 already known drug targets (both do have all 4 key properties) are listed. Table S2 lists all human signalog proteins and their drug target relevance scores.

Predicted signaling roles of human signalog proteins that are relevant in drug target discovery. The list of drug target proteins was downloaded from DrugBank [50]. The relevance score (for being a drug target) is the number of properties out of the following 4: membrane protein, enzymes, protein has a kinase domain, and disease-relatedness (see Fig. 6 and the main text for details). The list of human signalogs and their drug target relevance scores are available in Table S2.

In *C. elegans* and *D. melanogaster*, only 44 and 58 orthologs of human disease-related proteins (i.e., orthodisease proteins) have been annotated to signaling pathways, respectively [46]. In addition, we found pathway annotations for 10 (worm) and 9 (fly) additional orthodisease proteins among signalogs (see Table 3 and Table 4 for the lists of these proteins). For example, the human tyrosine-protein phosphatase SHP-2 protein has a single worm ortholog, *ptp-2*, which has not been annotated to any signaling pathway prior to our current study. On the other hand, the role of SHP-2 in multiple kinase pathways – including MAPK, JAK/STAT, and IGF – is well established. Thus, identifying novel signaling components *via* their orthologs may help future experimentation in model organisms and the description of the underlying disease mechanism.

**Table 3:** Orthodisease proteins among signalogs in *C. elegans*.

Signalog	Predicted pathway	Human ortholog(s)	Disease related to the human ortholog
ARR-1	EGF/MAPK, IGF/Insulin, WNT, Hedgehog, Notch,	S-arrestin (SAG)	Oguchi disease
LRP-1	Hedgehog, WNT	Low-density lipoprotein receptor-related protein 2 (LRP2)	Donnai-Barrow syndrome
PTC-1	Hedgehog	Protein patched homolog 1 and 2 (PTC1, PTC2)	Basal cell nevus syndrome and carcinoma,Holoprosencephaly7,Medulloblastoma
PTC-3	Hedgehog	Protein patched homolog 3 (PTC3)	Basal cell nevus syndrome and carcinoma,Holoprosencephaly7, Medulloblastoma
CRB-1	Notch	Crumbs homolog 1 and 2 (CRB1, CRB2)	Pigmented paravenous chorioretinal atrophy, Retinis pigmentosa
PTP-2	EGF/MAPK, IGF/Insulin, JAK/STAT	Tyrosine-protein phosphatase non-receptor type 11 (SHP-2)	Leopard syndrome, Noonan syndrome, Juvenile myelomonocytic leukemia
WRT-1	Hedgehog	Desert hedgehog protein (DHH) Indian hedgehog protein (IHH) Sonic hedgehog protein Precursor (SHH)	Gonadal dysgenesis, Brachydactyly, Acrocapitofemoral dysplasia, Holoprosencephaly, Solitary median maxillary central incisor, Polydactyly, Micropthalmia
TIG-3	TGF-β	Transforming growth factor beta-3 (TGFB3), Transforming growth factor beta-1 (TGFB1)	Arrhythmogenic ventricular dysplasia, Camurati-Engelmann disease
PAR-4	EGF/MAPK, WNT	Serine/threonine-protein kinase 11 (STK11)	Peutz-Jeghers syndrome, Testicular tumors
STA-1	JAK/STAT	Signal transducer and activator of transcription 1, 3, 4, 5b (STAT1, STAT3, STAT4, STAT5b)	Growth hormone insensitivity, Hyperimmunoglobulin E recurrent infection, Rheumatoid arthritis, Systemic lupus erythematosus, Atypical Mycobacteriosis

The *C. elegans* signalog proteins that have one or more human ortholog(s) related to a disease (orthodisease proteins). Note that predicted components of Hedgehog and JAK/STAT pathways are also present. The list of disease-related human proteins was downloaded from the Orthodisease database [46].

**Table 4:** Orthodisease proteins among signalogs in *D. melanogaster*.

Signalog	Predicted pathway	Human ortholog(s)	Disease related to the human ortholog
S6kII	EGF/MAPK	Ribosomal protein S6 kinase alpha-3 (RPS6KA3)	Coffin-Lowry syndrome
Rab23	Hedgehog	Ras-related protein 23 (RAB23)	Carpenter syndrome
smt3	TGF-β	Small ubiquitin-related modifier 4 (SUMO4)	Diabetes mellitus
bonus	TGF-β	Transcription intermediary factor 1 (TRIM24), E3 ubiquitin-protein ligase (TRIM33)	Thyroid carcinoma
lkb1	EGF/MAPK, WNT	Serine/threonine-protein kinase 11 (STK11)	Peutz-Jeghers syndrome, Testicular tumors
Pkc53E	EGF/MAPK	Protein kinase C gamma (PKCG)	Spinocerebellar ataxia
tws	EGF/MAPK	Serine/threonine-protein phosphatase 2A (PPP2R2B)	Spinocerebellar ataxia
Ror	TGF-β, WNT	Tyrosine-protein kinase transmembrane receptor (ROR2)	Brachydactyly, Robinow syndrome
ELP1	EGF/MAPK	Elongator complex protein 1 (IKBKAP)	Hereditary sensory neuropathy

The *D. melanogaster* signalog proteins that have one or more human ortholog(s) related to a disease (orthodisease proteins). The list of disease-related human proteins was downloaded from the Orthodisease database [46].

Finally, we tested the drug target relevance of human signalogs by examining 4 key drug-related properties: disease-relatedness, localization in the plasma membrane, enzymatic function, and kinase domain content (see Fig. 6a) [60]–[62]. To analyze the drug target related importance of the 73 human signalog proteins identified, we first selected 2 proteins (ANPRA [P16066] and CASK [O14936]) that have all 4 key properties (Fig. 6b). Both are established drug targets which supports the relevance of our analysis based on the 4 key properties. Our prediction suggests signaling roles for ANPRA and CASK in the WNT and EGF/MAPK pathways, respectively. We also predicted signaling roles for 3 additional proteins already used as drug targets (ABL2 [P42684], UGDH [O60701], and NDST1 [P52848]) (Fig. 6b). Novel pathway annotations of these drug targets are likely to provide additional details about their mechanisms of action, enrich therapeutic relevance, and warn of potential side effects. Following the top scoring set we analyzed those 2 proteins, INSRR [P14616] and MARK2 [Q7KZI7], that still have 3 of the 4 key properties, but are currently not used as drug targets and are not known to be disease-related. We predicted that INSRR functions in the IGF/Insulin pathway, while MARK2 functions in both the EGF/MAPK and Wnt pathways. Participation of MARK2 in more than one pathway increases its relevance for drug target discovery [22], [63]. Table 2 lists the predicted signaling roles and drug target-like properties for the most promising drug target candidates and the drugs targeting the already used drug targets. The complete list and drug target-like properties of signalogs can be found at http://signalink.org and in Table S2.

## Discussion

In our report we introduced a method for predicting novel signaling pathway components, signalogs, in 3 species, based on the signaling roles of their orthologs in other organisms. We identified altogether 253 signalog proteins in two model organisms – *C. elegans* and *D. melanogaster* – and humans. In addition, we developed and on-line tool allowing the users to predict signalogs and visualized known pathway data and predictions simultaneously with an ortholog network viewer. This viewer has distinctive features that can facilitate the interactive (user-defined) investigation of orthologous signaling networks, and it can visualize the predicted pathway components and their possible interactions, leading to the establishment of additional signalogs in later updates of the SignaLink database. The novelty of our predictions was verified by analyzing key properties, known pathway annotations, and already predicted interactions of signalogs. In *C. elegans*, we experimentally validated the signaling role of 6 predicted novel Notch pathway proteins. We anticipate that signalogs and, especially, orthodisease proteins of model organisms (see Table 3 and Table 4 and ref. [19]) can facilitate the design of novel, low cost primary drug screens with fewer tests in vertebrates. Our current study predicted signaling pathway memberships for 5 currently used drug target proteins and suggested 14 additional proteins that can be used as novel drug target candidates, based on their predicted pathway annotations and other properties (Table 2). Our predictions may (i) reveal novel therapeutic intervention points (e.g., the use of signalogs as novel targets to block specific pathways); (ii) suggest novel applications of current drugs to diseases, where the newly predicted signaling pathway of their target is relevant, and (iii) help to identify possible side effects of currently used drugs [59], [64], [65].

The identification of novel signaling components may have an impact on various fields of biology. Extended signaling annotation may allow a better explanation of unexpected mutant phenotypes by linking the altered (signalog) gene to a signaling pathway with known phenotypes. Biological research often focuses on altering the function(s) of a single selected protein. However, this may cause undesired dysregulation of a signaling pathway, interfere with multiple cellular processes and lead to pleiotropic effects. Therefore, constructing more comprehensive signaling networks and identifying novel signaling components can certainly improve the design and evaluation of experiments.

In the postgenomic era novel tools and methods are constantly needed to integrate genomic information with cellular processes. Current knowledge on signaling pathways is far from complete. ‘White spots’ can often be filled with the orthology-based transfer of ‘complete’ signaling system annotations between species, for example, by the method presented here. This method can be effectively used for the prediction of novel signaling proteins. The InParanoid database contains more than 100 eukaryotic genomes and its downloadable algorithm can be applied to other genomes as well, thus, orthology information is not a limiting factor for signalog predictions. Currently, signalog predictions for other genomes are limited mainly by the absence of proper signaling pathway data sources. Most importantly, the curation rules of databases should be uniform across all analysed organisms and signaling pathways. Such databases would be essential for signalog predictions both as seeds and as reference data sets. Unfortunately, for species not yet listed in the SignaLink database, current comprehensive signaling maps (databases) do not contain data curated with these guidelines in sufficient quantities that would allow the prediction of novel signaling components. The on-line tool was designed such that extensions can be added easily and we will include more species and pathways as soon as they become available. Based on the results, examples, and experimental work of this study, we believe that the predicted signaling pathway memberships (signalogs) will be a good source of functional hypotheses to be experimentally verified in all three investigated organisms.

## Supporting Information

Table S1 **Predicted signaling pathway member proteins (signalogs).** Protein names, species-specific identifiers, UniProt identifiers, and pathway annotations for all signalog proteins in the three investigated species.
(XLS)

Table S2 **List of human signalog proteins and their drug target relevances.** (1) Current drug target proteins and their signaling roles predicted in the current paper. (2) Predicted pathway annotations and further properties of proteins that are possible novel drug targets.
(XLS)

Text S1 **Experimental verification of signalog proteins.** Evaluation tables containing numerical experimental results and p-values quantifying the significance of experimental results.
(PDF)

## References

[1] Pires-daSilva A, Sommer RJ (2003). The evolution of signalling pathways in animal development.. Nat Rev Genet.

[2] Sakharkar MK, Sakharkar KR, Pervaiz S (2007). Druggability of human disease genes.. Int J Biochem Cell Biol.

[3] Beyer A, Bandyopadhyay S, Ideker T (2007). Integrating physical and genetic maps: from genomes to interaction networks.. Nat Rev Genet.

[4] Gabaldon T, Huynen MA (2004). Prediction of protein function and pathways in the genome era.. Cell Mol Life Sci.

[5] Kuzniar A, van Ham RC, Pongor S, Leunissen JA (2008). The quest for orthologs: finding the corresponding gene across genomes.. Trends Genet.

[6] Yan H, Venkatesan K, Beaver JE, Klitgord N, Yildirim MA (2010). A genome-wide gene function prediction resource for *Drosophila melanogaster*.. PLoS One.

[7] Costello JC, Dalkilic MM, Beason SM, Gehlhausen JR, Patwardhan R (2009). Gene networks in *Drosophila melanogaster*: integrating experimental data to predict gene function.. Genome Biol.

[8] Yellaboina S, Dudekula DB, Ko MS (2008). Prediction of evolutionarily conserved interologs in *Mus musculus*.. BMC Genomics.

[9] Storm CE, Sonnhammer EL (2003). Comprehensive analysis of orthologous protein domains using the HOPS database.. Genome Res.

[10] Salgado D, Gimenez G, Coulier F, Marcelle C (2008). COMPARE, a multi-organism system for cross-species data comparison and transfer of information.. Bioinformatics.

[11] Yu H, Luscombe NM, Lu HX, Zhu X, Xia Y (2004). Annotation transfer between genomes: protein-protein interologs and protein-DNA regulogs.. Genome Res.

[12] Persico M, Ceol A, Gavrila C, Hoffmann R, Florio A (2005). HomoMINT: an inferred human network based on orthology mapping of protein interactions discovered in model organisms.. BMC Bioinformatics.

[13] Cusick ME, Yu H, Smolyar A, Venkatesan K, Carvunis AR (2009). Literature-curated protein interaction datasets.. Nat Methods.

[14] Jensen LJ, Kuhn M, Stark M, Chaffron S, Creevey C (2009). STRING 8–a global view on proteins and their functional interactions in 630 organisms.. Nucleic Acids Res.

[15] Li D, Liu W, Liu Z, Wang J, Liu Q (2008). PRINCESS, a protein interaction confidence evaluation system with multiple data sources.. Mol Cell Proteomics.

[16] Brown KR, Jurisica I (2005). Online predicted human interaction database.. Bioinformatics.

[17] Kemmer D, Huang Y, Shah SP, Lim J, Brumm J (2005). Ulysses - an application for the projection of molecular interactions across species.. Genome Biol.

[18] Huang TW, Tien AC, Huang WS, Lee YC, Peng CL (2004). POINT: a database for the prediction of protein-protein interactions based on the orthologous interactome.. Bioinformatics.

[19] McGary KL, Park TJ, Woods JO, Cha HJ, Wallingford JB (2010). Systematic discovery of nonobvious human disease models through orthologous phenotypes.. Proc Natl Acad Sci U S A.

[20] Bauer-Mehren A, Furlong LI, Sanz F (2009). Pathway databases and tools for their exploitation: benefits, current limitations and challenges.. Mol Syst Biol.

[21] Joshi-Tope G, Gillespie M, Vastrik I, D'Eustachio P, Schmidt E (2005). Reactome: a knowledgebase of biological pathways.. Nucleic Acids Res.

[22] Korcsmaros T, Farkas IJ, Szalay MS, Rovo P, Fazekas D (2010). Uniformly curated signaling pathways reveal tissue-specific cross-talks and support drug target discovery.. Bioinformatics.

[23] Beltrao P, Serrano L (2007). Specificity and evolvability in eukaryotic protein interaction networks.. PLoS Comput Biol.

[24] Chaudhuri A, Chant J (2005). Protein-interaction mapping in search of effective drug targets.. Bioessays.

[25] Sergina NV, Rausch M, Wang D, Blair J, Hann B (2007). Escape from HER-family tyrosine kinase inhibitor therapy by the kinase-inactive HER3.. Nature.

[26] Bandyopadhyay S, Sharan R, Ideker T (2006). Systematic identification of functional orthologs based on protein network comparison.. Genome Res.

[27] Koonin EV (2005). Orthologs, paralogs, and evolutionary genomics.. Annu Rev Genet.

[28] Kelley BP, Yuan B, Lewitter F, Sharan R, Stockwell BR (2004). PathBLAST: a tool for alignment of protein interaction networks.. Nucleic Acids Res.

[29] Tatusov RL, Galperin MY, Natale DA, Koonin EV (2000). The COG database: a tool for genome-scale analysis of protein functions and evolution.. Nucleic Acids Res.

[30] Altschul SF, Madden TL, Schaffer AA, Zhang J, Zhang Z (1997). Gapped BLAST and PSI-BLAST: a new generation of protein database search programs.. Nucleic Acids Res.

[31] Berglund AC, Sjolund E, Ostlund G, Sonnhammer EL (2008). InParanoid 6: eukaryotic ortholog clusters with inparalogs.. Nucleic Acids Res.

[32] Chen H, Sharp BM (2004). Content-rich biological network constructed by mining PubMed abstracts.. BMC Bioinformatics.

[33] Fernandez JM, Hoffmann R, Valencia A (2007). iHOP web services.. Nucleic Acids Res.

[34] Boutet E, Lieberherr D, Tognolli M, Schneider M, Bairoch A (2007). UniProtKB/Swiss-Prot: The Manually Annotated Section of the UniProt KnowledgeBase.. Methods Mol Biol.

[35] Ashburner M, Ball CA, Blake JA, Botstein D, Butler H (2000). Gene ontology: tool for the unification of biology.. The Gene Ontology Consortium Nat Genet.

[36] Harris TW, Antoshechkin I, Bieri T, Blasiar D, Chan J (2010). WormBase: a comprehensive resource for nematode research.. Nucleic Acids Res.

[37] Drysdale R (2008). FlyBase: a database for the *Drosophila* research community.. Methods Mol Biol.

[38] Ogata H, Goto S, Sato K, Fujibuchi W, Bono H (1999). KEGG: Kyoto Encyclopedia of Genes and Genomes.. Nucleic Acids Res.

[39] Yu J, Pacifico S, Liu G, Finley RL (2008). DroID: the *Drosophila* Interactions Database, a comprehensive resource for annotated gene and protein interactions.. BMC Genomics.

[40] Simonis N, Rual JF, Carvunis AR, Tasan M, Lemmens I (2009). Empirically controlled mapping of the *Caenorhabditis elegans* protein-protein interactome network.. Nat Methods.

[41] Greenwald I (2005). LIN-12/Notch signaling in *C. elegans*.. WormBook.

[42] Brenner S (1974). The genetics of *Caenorhabditis elegans*.. Genetics.

[43] Kamath RS, Ahringer J (2003). Genome-wide RNAi screening in *Caenorhabditis elegans*.. Methods.

[44] Huang dW, Sherman BT, Lempicki RA (2009). Systematic and integrative analysis of large gene lists using DAVID bioinformatics resources.. Nat Protoc.

[45] Amberger J, Bocchini CA, Scott AF, Hamosh A (2009). McKusick's Online Mendelian Inheritance in Man (OMIM).. Nucleic Acids Res.

[46] O'Brien KP, Westerlund I, Sonnhammer EL (2004). OrthoDisease: a database of human disease orthologs.. Hum Mutat.

[47] Becker KG, Barnes KC, Bright TJ, Wang SA (2004). The genetic association database.. Nat Genet.

[48] Hunter S, Apweiler R, Attwood TK, Bairoch A, Bateman A (2009). InterPro: the integrative protein signature database.. Nucleic Acids Res.

[49] Harris MA, Clark J, Ireland A, Lomax J, Ashburner M (2004). The Gene Ontology (GO) database and informatics resource.. Nucleic Acids Res.

[50] Wishart DS (2008). DrugBank and its relevance to pharmacogenomics.. Pharmacogenomics.

[51] Kimble J, Simpson P (1997). The LIN-12/Notch signaling pathway and its regulation.. Annu Rev Cell Dev Biol.

[52] Bolos V, Grego-Bessa J, de la Pompa JL (2007). Notch signaling in development and cancer.. Endocr Rev.

[53] Takacs-Vellai K, Vellai T, Chen EB, Zhang Y, Guerry F (2007). Transcriptional control of Notch signaling by a HOX and a PBX/EXD protein during vulval development in *C. elegans*.. Dev Biol.

[54] Sternberg PW (2005). Vulval development.. WormBook.

[55] Szabo E, Hargitai B, Regos A, Tihanyi B, Barna J (2009). TRA-1/GLI controls the expression of the Hox gene lin-39 during *C. elegans* vulval development.. Dev Biol.

[56] Yoo AS, Bais C, Greenwald I (2004). Crosstalk between the EGFR and LIN-12/Notch pathways in *C. elegans* vulval development.. Science.

[57] Fay DS, Yochem J (2007). The SynMuv genes of *Caenorhabditis elegans* in vulval development and beyond.. Dev Biol.

[58] Gehlenborg N, O'Donoghue SI, Baliga NS, Goesmann A, Hibbs MA (2010). Visualization of omics data for systems biology.. Nat Methods.

[59] Berger SI, Iyengar R (2009). Network analyses in systems pharmacology.. Bioinformatics.

[60] Gao Z, Li H, Zhang H, Liu X, Kang L (2008). PDTD: a web-accessible protein database for drug target identification.. BMC Bioinformatics.

[61] Fabbro D, Ruetz S, Buchdunger E, Cowan-Jacob SW, Fendrich G (2002). Protein kinases as targets for anticancer agents: from inhibitors to useful drugs.. Pharmacol Ther.

[62] Yildirim MA, Goh KI, Cusick ME, Barabasi AL, Vidal M (2007). Drug-target network.. Nat Biotechnol.

[63] Hopkins AL (2008). Network pharmacology: the next paradigm in drug discovery.. Nat Chem Biol.

[64] Schadt EE, Friend SH, Shaywitz DA (2009). A network view of disease and compound screening.. Nat Rev Drug Discov.

[65] Wist AD, Berger SI, Iyengar R (2009). Systems pharmacology and genome medicine: a future perspective.. Genome Med.

