# Supplementary material for: Signalogs: Orthology-Based Identification of Novel Signaling Pathway Components in Three Metazoans
Source: PLoS One. 2011 May 3;6(5):e19240. doi: 10.1371/journal.pone.0019240 (PMC3086880; doi:10.1371/journal.pone.0019240)
Supplement: Text S1 — Experimental verification of signalog proteins. Evaluation tables containing numerical experimental results and p-values quantifying the significance of experimental results. (PDF) [file pone.0019240.s003.pdf]

Experimental validation of 6 signalog proteins

Here we provide a detailed statistics about the experiments verifying the predicted Notch pathway membership of the *C. elegans* genes: *aqp-6, D1009.3, nsh-1, num-1, F10D7.5,* and *crb-1*. For *aqp-6, crb-1, num-1,* and *D1009.3* we analyzed loss-of-function mutations treated with *lin-12* RNAi (Supplementary Table 1.), for *nsh-1* and *F10D7.5* we applied RNAi in *lin-12(gf)* mutant animals (Supplementary Table 2. and Supplementary Figure 1.)

		**Phenotype**			
**Genotype**	**RNAi**	**Normal vulva**	**Protruding vulva**	**Number of examined worms**	**P value**
N2	*lin-12*	82.8%	17.2%	320	–
aqp-6(tm2407)	*lin-12*	74.7%	25.3%	182	0.0298
crb-1(ok931)	*lin-12*	76.0%	24.0%	318	0.0359
num-1(ok433)	*lin-12*	70.9%	29.1%	182	0.0018
D1009.3(ok1349)	*lin-12*	70.0%	30.0%	163	0.0011

Supplementary Table 1. Summary of the phenotypes of loss-of-function mutants treated with *lin-12* RNAi. The *N2* genotype is the control experiment. Assuming statistically independent measurements the error of the control result (N2) is ~5%. P values were computed with Chi-square probes. Compared to the control experiment in all four cases the phenotype was significantly shifted from the normal vulva towards the protruding vulva (Pvl).

		**Phenotype**				
**Genotype**	**RNAi**	**Normal vulva**	**Multivulva**	**Number of vulvae (mean +/- std.dev.)**	**Number of examined worms**	**P value**
lin-12(gf)	-	7%	93%	3.27 +/- 0.19	118	–
lin-12(gf)	*nsh-1*	17%	83%	2.75 +/- 0.16	175	0.0024
lin-12(gf)	*F10D7.5*	19%	81%	2.75 +/- 0.17	176	0.0069

Supplementary Table 2. Summary of the phenotypes of *lin-12(gf)* mutants. P values were computed with Chi-square probes. In both tests (performed for *nsh-1* and *F10D7.5*) the number of vulvae is reduced with a high statistical significance compared to the control case.

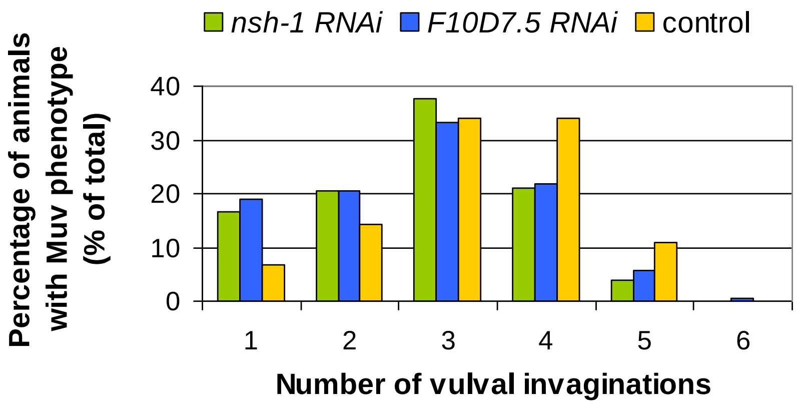

Supplementary Figure 1. Distribution of the number of vulvae in *lin-12(gf)* mutants. Observe that – compared to the *lin-12(gf)* control strain – both the *nsh-1* RNAi and *F10D7.5* RNAi animals were shifted towards smaller numbers of vulvae. Supplementary Table 2 displays averages of the data shown in this figure.
